# Exploring the *Staphylococcus aureus* host-pathogen interface using degradomic technologies

**DOI:** 10.1128/iai.00300-26

**Published:** 2026-10-08

**Authors:** Emilee M. Mustor, Lindsey N. Shaw

**Affiliations:** 1 Department of Molecular Biosciences, University of South Florida 7831, Tampa, Florida, USA; 2 Center for Antimicrobial Resistance, University of South Florida 7831, Tampa, Florida, USA; University of Pittsburgh, Pittsburgh, Pennsylvania, USA

**Keywords:** *Staphylococcus aureus*, degradomics, N-terminomics, proteases, pathodegradome

## Abstract

*Staphylococcus aureus* deploys an arsenal of secreted proteases to remodel the host environment during infection. While traditional substrate-by-substrate characterization has revealed critical host proteins under protease modulation, these conventional strategies cannot reveal the full scope of proteolytic activity occurring at the host-pathogen interface. Degradomic and N-terminomic technologies have begun to bridge this gap by enabling an unbiased, global mapping of protease-generated neo-N-termini across complex host tissues and cell proteomes. Recent work applying these tools to define discrete *S. aureus* protease pathodegradomes has propelled the field toward a system-level understanding of proteolytic activity. In this minireview, we highlight how terminal amine isotopic labeling of substrates and a newer methodology, terminal amine guanidination of substrates-charge reversal, have captured a deeper understanding of the V8 protease and how this enzyme can dismantle host immune defenses and engender disease. These techniques offer a powerful framework for the future in characterizing the substrate repertoires of other *S. aureus* secreted proteases, such as aureolysin, staphopain A and B, and the Spls. Furthermore, these advances demonstrate the applicability of degradomic profiling to diverse infection niches, revealing previously unrecognized roles for proteolytic activity in varying host contexts. Finally, we consider how this comprehensive understanding of *S. aureus* protease activity can inform clinical applications such as protease inhibitor design, host-directed therapies, and pathodegradome-based diagnostics.

## INTRODUCTION

Across the evolutionary history of host-pathogen interaction, proteolysis represents a conserved and potent mechanism of host manipulation, leveraged by microbial pathogens spanning multiple kingdoms of life (1–6). The irreversible nature of hydrolyzing peptide bonds renders proteolysis a decisive virulence strategy, ensuring profound consequences for both the host and invading pathogen (7). Indeed, the effects of proteolysis are far-reaching, where cleavage of an individual substrate can induce dysfunction across myriad biological networks (7, 8). On one hand, infectious organisms can trigger uncontrolled protein degradation, often leading to heightened inflammation, followed by morbidity and, in certain cases, mortality (8, 9). Conversely, precision proteolysis, where cleavage occurs in a highly specific manner, provides a level of pathogen sophistication that shines a light on meaningful events that occur at the host-pathogen interface (7–9). For dangerous pathogens like *Staphylococcus aureus*, proteolysis is a defining feature of bacterial virulence, driven primarily by its diverse repertoire of secreted proteases that target multiple dimensions of the host immune response (10–12). These proteases include the metalloprotease aureolysin, the cysteine proteases staphopain A (ScpA) and staphopain B (SspB), as well as the serine protease V8 (SspA) (10, 11). *S. aureus* strains can also produce up to nine serine-protease-like enzymes, known as the Spls, depending on their specific sequence type (13). These virulence-impacting enzymes act as precise molecular effectors at the host-pathogen interface (14), disrupting protective host mechanisms such as the complement system, immunoglobulins, host proteases, and antimicrobial peptides, with remarkable specificity (15–18). As such, these virulence factors are fundamental to *S. aureus* pathogenic success, creating a favorable environment within the host that promotes bacterial survival, dissemination, and persistence. Understanding the full scope of their activity and defining their substrate libraries thus represent a fundamental step toward unraveling the molecular basis of protease-driven immune evasion in *S. aureus*. Understanding or elucidating protease activity remains complicated due to gaps in our knowledge regarding their expression levels, spatiotemporal distribution, turnover, and more, all of which greatly influence cleavage activity in physiologically relevant contexts (19). Historically, the evaluation of protease activity for *S. aureus* and other pathogens has relied on two principal experimental pillars: gene deletion approaches for analyzing their contribution to virulence *in vivo* and *in vitro* biochemical assays that allow for one-at-a-time substrate identification. While these studies have yielded valuable insights, they have fundamental limitations that make accurate understanding of these enzymes difficult to achieve. Targeted gene deletion strategies are complicated by the functional redundancy of the secreted proteases, where commonalities in substrates have been observed (20). This may explain contradictory animal studies, where protease deletion has resulted in attenuated virulence in certain cases, yet in others, the opposite effect or the absence of any phenotype at all has been highlighted (10, 11, 21–25). *In vitro* substrate assays, though informative, are typically conducted on purified proteins or using antibodies against a specific target of interest (15, 18, 20, 26). These substrate assays thus operate within the confines of pre-existing knowledge, often leaving novel substrates or the broader picture overlooked. As such, these methods remain limited in their capacity to provide a comprehensive and unbiased picture of protease substrate landscapes, as they negate the biological complexity of real infection environments. With these challenges at the forefront of protease substrate characterization, the emergence of degradomic technologies has begun to bridge this gap by enabling comprehensive mapping of cleavage events within physiologically relevant contexts (7, 27). Degradomics employs mass spectrometry-based profiling of native as well as new or “neo” N- or C-termini that have been generated following proteolytic cleavage (28, 29). The application of degradomics to *S. aureus* research is a critical step forward, enabling comprehensive and high-throughput mapping of protease activity in a manner that classical approaches cannot achieve. For *S. aureus*, where multiple proteases interact with a complex and dynamic host proteome, exposing novel substrates and immune evasion mechanisms can significantly advance our fundamental understanding of pathogenesis (12, 30). Critically, the ability to resolve the degradomic landscape across diverse host niches occupied by *S. aureus* further amplifies the power of degradomics, enabling context-specific interrogation of host-protease interactions. In this minireview, we survey the extracellular protease arsenal of *Staphylococcus aureus*, examining individual cleavage specificity and current knowledge on protease substrate targets, highlighting functional redundancy that has long complicated their study. We explore how the emergence of degradomic technologies is redefining our understanding of protease-mediated host manipulation. Collectively, we present a comprehensive consideration of how precision proteolysis may shape the outcome of *S. aureus* infection, together with a view into what the degradomic future may hold for antivirulence therapeutic strategies.

## INSIGHTS INTO CLEAVAGE SPECIFICITY

Proteolysis is the hydrolysis of peptide bonds, where specificity governs the substrates that are targeted, the precise sites at which cleavage occurs, and ultimately, the functional consequences that follow (7). Each *S. aureus* extracellular protease exhibits a distinct substrate cleavage specificity, collectively enabling the bacterium to target a broad range of host proteins (12, 30). SspA/V8 selectively hydrolyzes peptide bonds C-terminal to glutamic acid residues, with aspartate residues representing a secondary but less frequent cleavage site (31). Aureolysin cleaves peptide bonds positioned N-terminally to bulky hydrophobic residues, with leucine, valine, alanine, isoleucine, phenylalanine, and tyrosine representing prominent examples of favored substrates (32–34). The cysteine proteases of the *S. aureus* proteolytic arsenal, ScpA and SspB, contribute an additional layer of proteolytic activity and share a broadly conserved cleavage motif of P3-P2-Gly-*-Ser(Ala) (35). However, substrate discrimination between ScpA and SspB is primarily governed by differing preferences at the P2 position (35). Staphopain A typically prefers hydrophobic residues like leucine at P2, whereas staphopain B favors bulky aromatic residues such as phenylalanine or tyrosine (35, 36). However, SspB further diverges from canonical papain-like proteases and exhibits an unconventional specificity for serine or threonine, C-terminal to the P1’ substrate bond (36). Beyond the four major extracellular proteases, the serine protease-like enzymes (Spls) represent the most recent group of identified proteases, and their characterization is still ongoing. SplA, SplB, and SplD are distinguished by their exceptionally narrow substrate recognition sites, found to be a four- to five-amino acid consensus sequence for each protease (37–40). SplA recognizes the motif (W/Y)-L-Y-*-(T/S) (38); SplB cleaves within the sequence W-E-L-Q-* (37); and SplD targets R-(Y/W)-(P/L)-(T/L/I/V)-*-S (39). This pattern of highly constrained substrate recognition extends to the more recently studied Spl members, SplE and SplF, where SplE hydrolyzes peptide bonds at (L/V)-(W/Y/F)-L-(H/Q)-*G (41), and SplF identifies (Y/F)-(M/Y/H)-(L/M)*S (42). In contrast, the substrate specificity of SplC remains to be fully elucidated, and as such, its precise role within the context of *S. aureus* host-pathogen interaction remains unclear. Taken together, the diverse and highy specialized substrate specificities of the *S. aureus* extracellular proteases collectively highlight the remarkable precision of these factors and the central role they play in the overall virulence and pathogenic success of this bacterium.

## OLD APPROACHES PAINT A PATCHWORK OF SUBSTRATES FOR STAPHYLOCOCCAL PROTEASES

**Fig 1:**
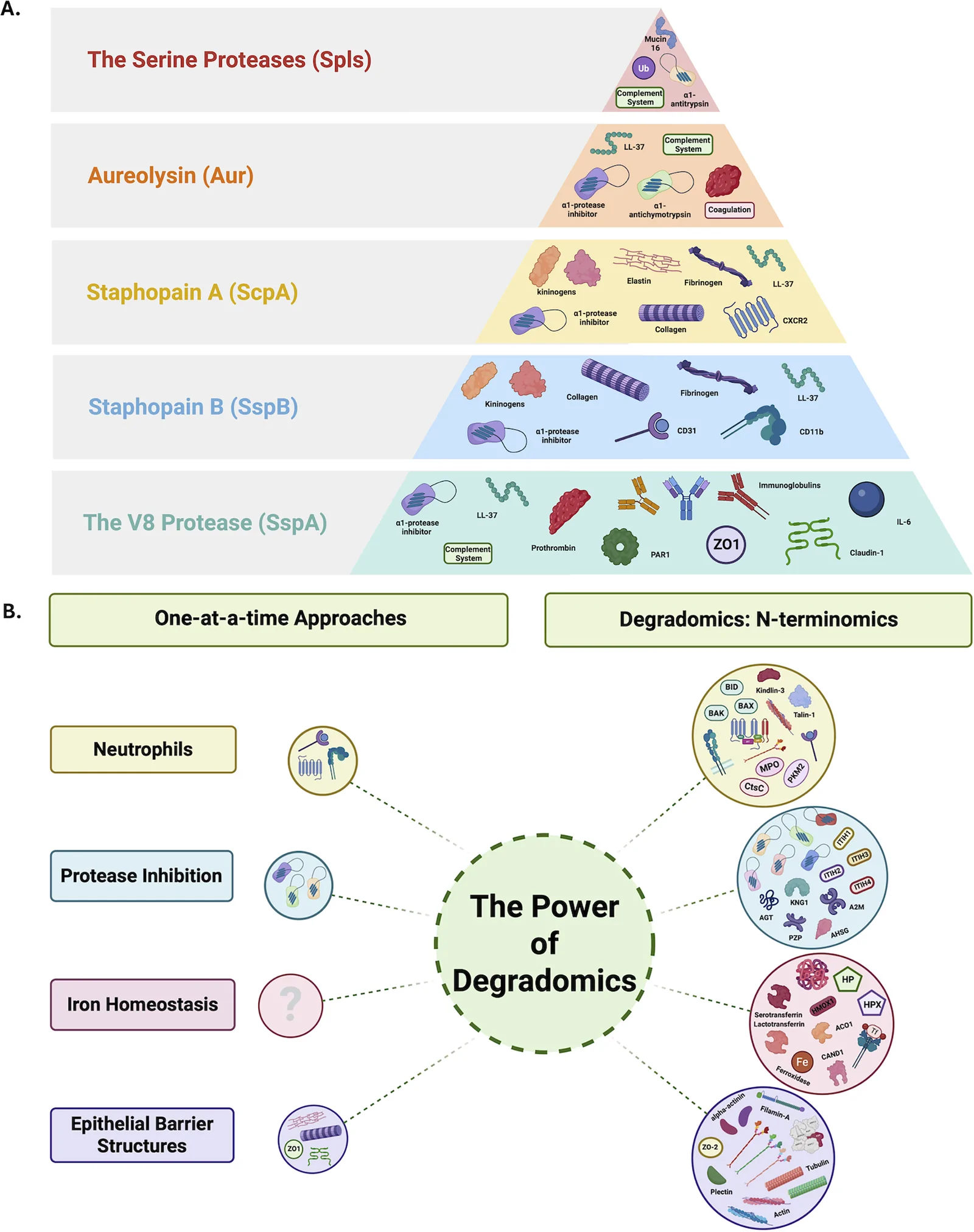
The power of degradomics versus canonical approaches to protease substrate identification. (**A**) Shown are host substrates identified using one-at-a-time approaches for the major secreted proteases of *S. aureus*: the serine protease-like enzymes (SpIs), aureolysin (Aur), staphopain A (ScpA), staphopain B (SspB), and the serine protease V8 (SspA). Substrates span immune effectors (complement components, immunoglobulins, LL-37, IL-6), protease inhibitors (α1-antitrypsin, α1-antichymotrypsin, α1-protease inhibitor), extracellular matrix and coagulation factors (collagen, elastin, fibrinogen, prothrombin), cell surface receptors and junction proteins (chemokine receptor 2 [CXCR2], CD31, CD11b, PAR1, ZO1, claudin-1), and kininogens. (**B**) Degradomic approaches reveal a substantially broader substrate landscape than one-at-a-time methods across four biological themes relevant to *S. aureus* pathogenesis: neutrophil biology, protease inhibition, iron homeostasis, and epithelial barrier structures. For each biological process, conventional approaches identify only a small handful of known substrates (left circles), whereas degradomic profiling, specifically N-terminomics, captures a much larger and more diverse set of protease targets (right circles). Notably, no one-at-a-time substrates have been reported for iron homeostasis, highlighted by the question mark, underscoring a gap that degradomics is now starting to fill.

Despite decades of targeted biochemical investigation, a less than complete picture of protease function has emerged, reflecting a patchwork of individually identified targets that misses the bigger picture of comprehensive protease characterization at the host-pathogen interface (Fig. 1A).

Aureolysin contributes to immune evasion through cleavage of the antimicrobial peptide LL-37, neutralization of the host protease inhibitors α1-protease inhibitor and α1-antichymotrypsin, and manipulation of the coagulation and complement systems (18, 20, 43, 44). Aureolysin also cleaves complement C3 to liberate C3a and C3b, dampening neutrophil responses and minimizing opsonization (45). Furthermore, this protease can degrade complement factor C5 to release the anaphylatoxin C5a, though this activity appears concentration dependent and is contested in the literature (15, 45). The cysteine proteases of *S. aureus*, ScpA and SspB, can cleave kininogen to release bradykinin, causing vascular leakage (VL), where SspB enhances ScpA’s release of bradykinin. However, when these two proteases work in concert, they also release a unique kinin that can cause VL (25). These proteases can further cause tissue injury by cleaving connective host proteins such as elastin by ScpA and collagen by both ScpA and SspB, as well as fibrinogen (46, 47). They also share the antimicrobial peptide LL-37 and the coagulation substrate α1-protease inhibitor as targets with other members of the protease arsenal (20, 48). In terms of neutrophil function, staphopain A can cleave chemokine receptor 2 on the leukocyte cell surface, causing diminished neutrophil activation and recruitment (26). Likewise, neutrophil activity was found to be impaired by staphopain B-induced cleavage of both platelet endothelial adhesion molecule-1 (CD31) and CD11b, also found on the neutrophil surface (49, 50). The Spls are the least characterized protease group, and the difficulty of substrate capture here is compounded by the sheer number of individual members with distinct specificities. Currently confirmed substrates for the Spls include complement factors C3, C4, C5, C6, C7, C8, and C9 for SplB (51). Mucin-16 is cleaved by SplA, where this cleavage event is thought to promote *S. aureus* virulence in pneumonia infections (24). With respect to neutrophil function, only a single substrate has thus far been identified that connects Spl activity to the modulation of neutrophil activity and responses: alpha-1 antitrypsin (AAT) (52). This serine protease inhibitor was found to be cleaved by SplD, SplE, and SplF in its reactive center loop, generating a 36-aa fragment (52). Finally, for defined substrates, a recent study found SplA and SplB to cleave ubiquitin or ubiquitin-like enzymes, with the suggested likely outcome of manipulating immune cell signaling pathways (53). In terms of disease contribution, SplD has been linked to allergic asthma, where this protease can trigger the production of IgE antibodies as well as activate TH2 response, resulting in airway inflammation (54). Moreover, many olfactory receptors contain consensus sequences pointing to them as SplB substrates (37).

The V8 protease is perhaps the most extensively characterized extracellular protease, with the broadest range of known substrates. Shared targets with aureolysin include α_1_-proteinase inhibitor (20), complement factors C3, C5, and others (45), as well as prothrombin (55) and LL-37; however, here, this cleavage has no functional consequence (18). V8 is also well known to cleave multiple classes of immunoglobulins, including IgG, IgM, and IgA, targeting the Fc region of these antibody classes, resulting in partial loss of antigen recognition and impaired interaction with immune effector cells (16). This protease can also directly influence host sensory pathways, possessing a unique role in inducing itch through cleavage and activation of PAR1 on mouse and human sensory neurons (56–58). While *in vitro* cleavage of claudin-1 has been confirmed, degradation or rearrangement has only been hypothesized for ZO-1 (57, 58). Similarly, ELISA-based evidence of reduced IL-6 production following V8 exposure is suggestive of proteolytic processing but does not define a cleavage site (58).

## DEGRADOMIC TECHNOLOGIES: A FRAMEWORK FOR EXPLORING THE HOST-PATHOGEN INTERFACE

A major roadblock in the field of protease biology is that many proteolytic events are detected without resolution of the precise cleavage site, leaving gaps in our mechanistic understanding of how these enzymes work. Furthermore, holding true for V8 and almost all the *S. aureus* proteases, *in vivo* studies demonstrating a protease-related phenotype typically fail to link protease effects with any known host protein targets (54, 59, 60). However, with the implementation of degradomic technologies, we are now revealing a scale of host manipulation that classical approaches simply cannot achieve.

**Fig 2:**
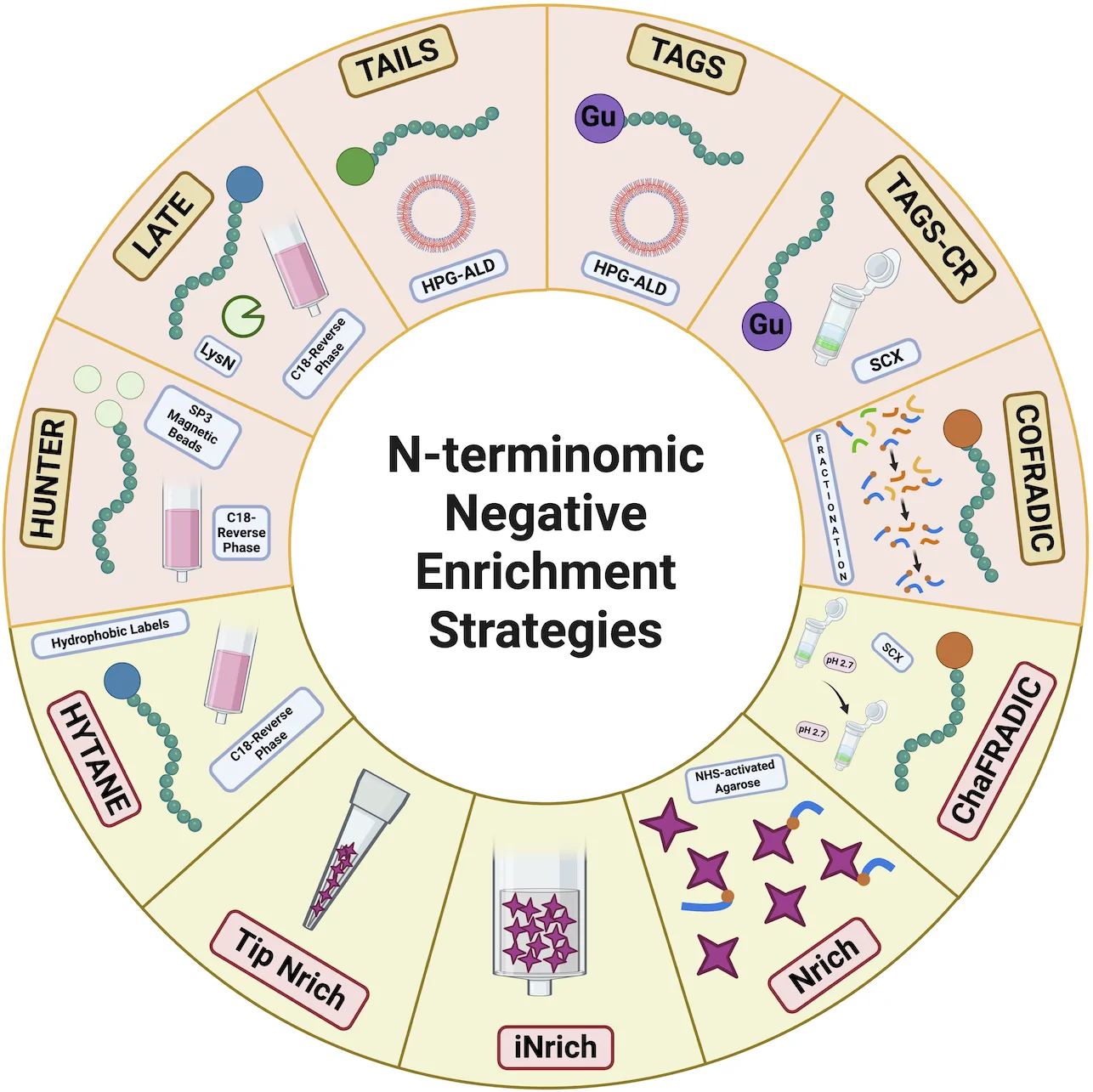
N-terminomic negative enrichment strategies. The principal approach, CHOFRADIC, uses two steps of chromatographic separation, while ChaFRADIC uses two steps of strong cation exchange for enrichment. Nrich, iNrich, and Tip Nrich use NHS-activated agarose in different capacities for capture and depletion of internal tryptic and GluC peptides, allowing for isolation of tagged N-termini of interest using filter-aided sample preparation. HYTANE, HUNTER, and LATE are negative enrichment strategies that implement hydrophobic labeling to remove background peptides. HYTANE increases the hydrophobicity of internal peptides with bis-hexadecanal followed by reverse-phase separation for isolation. HUNTER utilizes solid-phase enhanced sample preparation (SP3) beads for labeling and enrichment of N-termini. The LATE methodology utilizes LysN for digestion of peptides; next, ɑ-amines are demethylated, and lysine residues are labeled with undecanal, allowing for depletion using C18 reverse-phase material. TAILS combines isotopic labeling with enrichment using the water-soluble HPG-ALD polymer to remove background peptides. TAGS makes use of guanidine labeling while also combining this with HPG-ALD-mediated N-terminome enrichment. TAGS-CR combines guanidination of N-termini of interest with strong cation exchange (SCX) for N-terminome enrichment.

When proteases cleave their desired targets, this gives rise to a newly generated or neo-N- and C-termini. Appropriately, degradomic methods to identify these substrate products have been distinguished as either N-terminomics or C-terminomics, respectively (61, 62). Two principal objectives typically form the basis of these degradomic techniques: labeling of degradation products and enrichment strategies (29). How effectively these are achieved, however, differs considerably between N- and C-terminomic approaches. C-terminomics captures the liberated carboxyl terminus following cleavage. However, the poor reactivity of the carboxyl groups, together with reduced stability of the neo-C-termini, presents challenges for enrichment, ultimately resulting in limited adoption in the field of degradomics (62, 63). N-terminomics, by contrast, is the preferred technique to capture proteolysis in biological systems due to the high reactivity of the newly generated primary amine group that permits labeling for enrichment and downstream identification (28). Currently, two enrichment strategies exist, positive and negative. However, with the need for highly specialized reagents for positive selection that may also demonstrate bias when labeling the N-termini, negative methods have come to be preferred (Fig. 2) (27, 64)

Negative enrichment strategies offer a subtractive approach whereby all pre-existing protein N-termini are chemically blocked prior to protease treatment, such that depletion of background peptides allows for isolation of neo- and native N-termini for downstream identification (28). In this context, negative enrichment has the added benefit of capturing endogenously modified N-termini, providing broader insight into the N-terminal landscape via identification of alternative translation sites and thus possible protein variants (65, 66). Two principal negative approaches have been developed: combined fractional diagonal chromatography and charge-based enrichment (COFRADIC), one of the earliest methods, and terminal amine isotopic labeling of substrates (TAILS), which has since come to predominate the field of degradomics (67, 68). While COFRADIC was a major breakthrough for negative selection in degradomics, its implementation is typically less frequent because of its labor-intensive workflow, low throughput, requirement for significant sample material, and complexity in deciphering data (29). An alternative method known as ChaFRADIC was developed to mitigate certain limitations; however, it still remains technically demanding and consequently has not seen widespread use (69). TAILS, on the other hand, has become widespread precisely because it addresses many of the constraints seen in COFRADIC and its successor methodology (68). Chris Overall and colleagues developed this method, which combines isotopic blocking of pre-existing N-termini with HPG-ALD polymer-based depletion of internal tryptic peptides (68). This allows for the selective recovery of neo- and native N-termini, and furthermore, effective quantitation and multiplexing can be achieved as the distinct isotopic labels, such as TMT or iTRAQ, introduce mass differences that allow multiple samples to be pooled and comparatively analyzed via LC/MS-MS (68). Alternatives to dimethyl labeling do exist, such as terminal amine guanidination of substrates (TAGS) (70). However, a major limitation of both the TAILS and TAGS workflows is the use of the aldehyde-based polymer that incurs significant cost and can also be unstable. To circumvent this, negative enrichment has seen the utilization of different materials for internal peptide capture, for example, phospho-tag and titanium oxide-based depletion, sulfhydryl-based capture with iron(III) oxide microspheres, and NHS-activated agarose-based methods, including Nrich, iNrich, and TipNrich (71–75). Beyond these well-established chemistries for negative enrichment, hydrophobic labeling has gained traction, whereby internal tryptic peptides are labeled with fatty aldehydes and subsequently depleted using commercially available reverse-phase resins (76–78). HYTANE, HUNTER, and LATE are all examples using these core principles, each with their own individual strengths and weaknesses (76–78). Collectively, these strategies reflect not only the preference for negative enrichment over positive methods but also the rapidly expanding nature of degradomic techniques. Overall, the numerous approaches described herein reflect a field of study that is rapidly advancing, with an expanding suite of sensitive and accessible workflows now available for comprehensive capture of the N-terminome.

## DEGRADOMICS AND STAPHYLOCOCCAL PATHOGENESIS: A NEW FRONTIER

**Fig 3:**
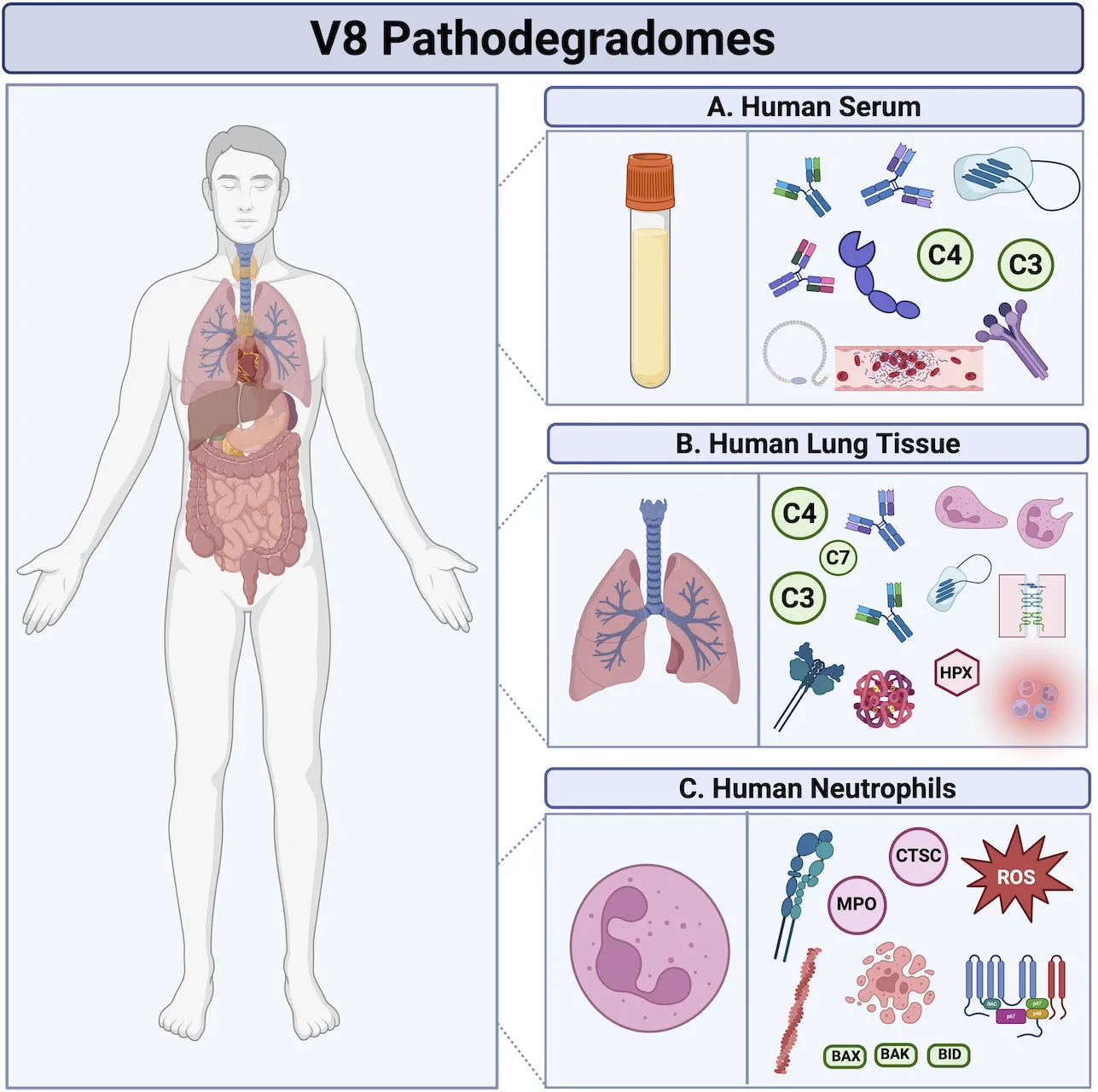
Current V8 pathodegradomes. Degradomic profiling has revealed the substrate repertoires of the V8 protease in various infection-relevant niches. TAILS revealed substrates for the V8 protease in (**A**) human serum, highlighting immunoglobulins, clotting factors, complement components, and serine protease inhibitors as host targets. TAGS-CR captured host targets for the V8 protease in (**B**) human lung tissue, capturing immunoglobulins, complement factors, neutrophil proteins, and epithelial barrier molecules as targets, as well as those involved in iron homeostasis and inflammation. TAGS-CR was also used to profile V8 activity in (**C**) human neutrophils, showing integrins, actin molecules, azurophilic granules, reactive oxygen species precursors, and apoptotic proteins as V8 substrates. The power of degradomics lies in the ability to apply these approaches to many different infection-relevant substrates or niches, leaving potential for future studies to continue to unravel the contribution of this virulence enzyme to *S. aureus* pathogenesis.

In *Staphylococcus aureus*, N-terminomics has gained traction, where a number of studies have begun to investigate proteolysis at the host-pathogen interface. For example, an N-terminomic pipeline was applied to map cell surface processing events targeting the moonlighting protein elongation factor (Tu) (79). Moreover, proteolytic profiling of vegetative growths on the heart valves of patients suffering from *S. aureus*-induced endocarditis was performed using the TAILS method (80). However, it is important to note that these studies cannot attribute any captured cleavage events to a distinct protease. To address this gap directly, our group has pioneered a new wave of degradomic investigations where global substrate profiling has been applied to infection-relevant niches that systematically define substrate repertoires of individual secreted proteases (17, 81, 82). These studies have honed in on the V8 protease of *S. aureus* in particular, to specifically explore this enzyme’s contribution to infection by identifying its immune evasion tactics (Fig. 3). One of the key features that makes V8 so useful in such studies is its strict substrate specificity, which provides supporting validation for cleavage events. TAILS was first utilized by Frey et al. (17) to profile V8 substrates in human serum. This study was able to identify a plethora of different host processes and fundamental pathways being disrupted by the proteolytic action of V8, attributable to the protease-induced degradation of approximately 90 host proteins (17). Major themes emerged in this data set, such as the targeting of host protease inhibitors by the V8 protease, together with additional substrates identified within the complement system and clotting cascade (17). It is important to note here that cleavage of these proteins occurred in a highly specific manner, where, for example, protease inhibitors were degraded in their reactive center loops or bait region domains, and complement factor cleavage occurred in active sites or functional interaction domains (17). Beyond these targets, V8 was also shown to cleave immunoglobulins and other multiregulatory proteins involved in the inflammatory response in human serum (17). Comprehensive substrate capture at this level highlights the power of global N-terminomics to resolve protease-driven host remodeling at an unprecedented resolution (17). A key strength of global N-terminomics lies in its applicability to diverse host niches, making it particularly well suited to the study of *S. aureus*, a pathogen capable of colonizing nearly every region of the human body, engendering a wide spectrum of disease types (7, 83, 84). In keeping with this, our more recent work profiled V8 substrates in human neutrophils as well as human lung tissue (81, 82). However, in contrast to the human serum study, these targets were revealed using a newly developed method of N-terminomics known as terminal amine guanidination of substrates-charge reversal (TAGS-CR) (81). TAGS-CR combines guanidination as the labeling strategy for tagging lysine ε-amines and N-terminal α-amines, with strong cation exchange (SCX) as the negative enrichment strategy for isolation of N-termini (70, 81, 85). Guanidination allows for a more effective tryptic digestion of guanidinyllysine, given its similarity in structure to arginine, where trypsin is also known to cleave (70). This is in comparison to traditional labeling approaches such as dimethyl or acetyl labeling, where cleavage is hindered due to steric bulk or charge alterations of the residue (70). Guanidination also improves ionization efficiency as guanidine has a greater propensity to acquire a proton (70). In terms of negative enrichment, strong cation exchange is a simplified approach that allows for the removal of internal tryptic peptides, rather than using the inefficient HPG-ALD polymer as seen in the traditional TAGS method (70, 81, 85). Sulfonation conjugates a negative charge to these unlabeled internal peptides, whereby they can no longer bind to the negatively charged SCX matrix, leaving behind labeled N-termini of interest for downstream MS/MS identification (81, 85). Taking these factors into account, TAGS-CR offers improved sensitivity and N-terminome coverage in comparison to the TAILS approach.

In human neutrophils, TAGS-CR captured approximately 350 V8 substrates, revealing how this protease adopts a broad and multifaceted strategy to disable neutrophil function (81). Cleaved targets spanned proteins essential to neutrophil migration and adhesion, degranulation, reactive oxygen species production, phagocytosis, and apoptotic regulation (81). These collectively represent critical immune cell processes that provide host defense against invading pathogens like *S. aureus* and enable efficient pathogen clearance (86). At the molecular level, neutrophil migration can be disrupted via V8 cleavage of ICAM3, GNAI2, RHOA, RAC2, MSN, and ITGAL, among many other protein targets identified in this pathway (81). Our study also highlights the striking specificity by which V8 cleaves, targeting active sites or functional domains, as in the case of ITGAL, where this protease cleaves in the beta propeller domain necessary for ligand binding (81). Furthermore, this method of global substrate characterization also sheds light on the sophisticated nature of protease activity, where cleavage not only may result in degradation but also actually strategically activate host proteins (81). This can be seen in the case of apoptotic proteins BAX, BAK, and BID, where V8 cleavage appears to result in structural rearrangement, and consequently downstream activation (81). This proteolytic event was hypothesized to promote dissemination, a role previously recognized for the *S. aureus* proteases (12, 81).

In further work using human lung tissue, TAGS-CR continued to reinforce the notion of V8 as a critical disruptor of host immune defense (82). This study captured a library of substrates that, while partially overlapping with the neutrophil pathodegradome, revealed new and notably niche-specific dimensions of V8’s proteolytic activity (82). Shared between both studies, important neutrophil proteins were identified with roles in intracellular defense, phagocytosis, and migration, such as PKM2, calreticulin, and kindlin-3. Speaking yet again to the specificity of the V8 protease, proteolysis of PKM2 was found to occur in the A domain across both studies, which is important because the interface of the A and B domains constitutes the binding pocket of this protein (87). Moreover, kindlin-3 cleavage by V8 was also found to occur at the exact same amino acid position in both settings, speaking to just how precise and consistent protease activity is, even in different biological niches (81, 82). Interestingly, alongside shared targets, our lung study also captured a complementary dimension of V8 activity, where this protease was found to cleave the interacting partner, ICAM-1, of the neutrophil V8 substrate ITGAL (81, 82). This suggests that V8 does not simply target proteins in isolation but rather systematically dismantles protein complexes by targeting multiple interacting components. This notion is further supported by V8’s interaction with the NADPH oxidase complex across both studies, where multiple and varying subunits were identified as substrates in each setting. Our human lung pathodegradome went on to reveal previously unknown substrates through which V8 drives barrier dysfunction, a pathogenic tactic long associated with this protease (58, 59). The identification of numerous barrier proteins as substrates provides, for the first time, a clearer picture of the molecular basis underpinning this phenomenon. TAGS-CR was also able to characterize new features of V8 protease activity via capture of substrates involved in inflammation and iron homeostasis (82). Toward this latter finding, iron-complexed proteins were cleaved that would potentially liberate iron for uptake by *S. aureus*, an important ion needed to survive and thrive during infection (88). Regarding inflammation, V8-mediated degradation of inflammatory substrates was suggested to induce a proinflammatory response (82). Lastly, a defining and novel feature of our study was the discovery of several substrates with established roles in lung-specific antibacterial defense that are cleaved by V8, identifying a niche-specific component of the human lung V8 pathodegradome. Given that each infectious niche presents immunological challenges, this work represents a critical step toward a more complete and context-dependent understanding of V8-driven immune dysregulation.

## WHAT DOES THE DEGRADOMIC FUTURE HOLD FOR *S. AUREUS* BIOLOGY

The degradomic studies described herein present foundational work, demonstrating how global substrate profiling can translate into a deeper mechanistic understanding of *S. aureus* proteases and their role in remodeling the host immune landscape (Fig. 1B). However, these pioneering studies have only now begun to scratch the surface of what N-terminomic and degradomic technologies can reveal as it relates to *S. aureus* pathogenesis. The substrate repertoires of several key proteases remain uncharacterized, leaving important questions about their individual contributions to immune evasion, tissue remodeling, and disease progression. Moreover, the functional redundancy of the *S. aureus* protease network points to a far greater level of coordinated proteolytic activity that degradomic studies have only now begun to shine light on. Another gap in the knowledge at the forefront of protease-mediated host-pathogen interplay is the contextual specificity of cleavage events across diverse host niches. *S. aureus* is a remarkably versatile human pathogen, thriving in almost every part of the human body, and with this comes specific environmental conditions, host cell populations, and immune pressures that may shift protease responses. While the TAGS-CR human lung study did showcase context-dependent protease activity, the field requires a much broader application of degradomics across clinically relevant infection models in order to see the complete picture. In this regard, more sophisticated experimental models are needed to recapitulate the infection environment and its dynamics in order to garner a more meaningful look at the proteolytic landscape. This speaks to not only *in vivo* degradomic approaches that can resolve proteolytic events as they occur within living tissues but also new and emerging techniques like spatial proteomics. *In vivo* degradomics is not without its challenges, however, given the inherent complexity of living systems, together with the difficulties of attributing specific cleavage events to individual proteases. Consequently, methodological progress in this area will be critical for advancing this newly burgeoning field. New technologies like the Microscoop, developed by Syncell (https://www.syncell.com/microscoop/), which performs *in situ* photo-biotinylation of proteins within a specific user-defined region of interest of an organelle, cell, or organism, represent a powerful new framework for protein resolution. The application of technology such as this to the field of degradomics could provide unparalleled insight into protease substrate landscapes, adding a layer of spatially resolved biological relevance that has yet to be captured. Finally, functional validation of identified substrates and cleavage events may be considered one of the most important next steps in translating degradomic discoveries into impactful biological and therapeutic insights. The global and unbiased nature of degradomic studies, a major strength of these methods, is also accompanied by the challenges of identifying biologically significant events that lead to tangible effects within the host. Indeed, robust strategies are necessary to determine the particular substrates that, when cleaved, produce the most significant disruption to host networks and ultimately influence infection outcomes. For pathogens like *S. aureus*, functional insight into protease-substrate interactions may present the most compelling therapeutic targets as an alternative to antibiotic treatment strategies. This may especially be useful in the current era of rising resistance in bacteria against these classes of drugs (89). It is precisely this clinical promise of degradomics that makes closing these knowledge gaps critical for *S. aureus* research. The major extracellular proteases produced by *S. aureus* are among the most consequential virulence factors for infection and yet are significantly underexploited in terms of therapeutic intervention. In this regard, several avenues exist for converting degradomic knowledge into clinical impact. One example is the use of protease inhibitors that can be designed using knowledge of substrate specificities and cleavage site architecture. Capturing protease cleavage sites, as demonstrated in the work on V8, provides a highly nuanced insight into protease substrate specificity that can drive the design of these molecules. With this knowledge, engineered decoy substrates may be developed that competitively occupy protease active sites and can serve to attenuate proteolysis. The use of protease inhibitors has already gained traction, for example, with aureolysin and the design of a modified therapeutic protein based on the insect metallopeptidase inhibitor (IMPI) from *Galleria mellonella* (90). Various IMPI mutants were developed to competitively block aureolysin activity, demonstrating this as an achievable therapeutic avenue (90). While these inhibitor molecules were not based on degradomic data, they could be guided and improved if the power of global substrate profiling was applied to aureolysin. In addition to competitive inhibition, protease inhibitors can also be developed to elicit irreversible binding and form strong covalent bonds with the catalytic residues within protease active sites. This strategy has been successfully developed in other infectious disease contexts, such as with HIV and the use of nonhydrolyzable hydroxyethylene and hydroxyethylamine isosteres in protease inhibitor molecules (91). Beyond inhibitor design, degradomic data sets could also open the doors to host-directed therapies. Here is where addressing the functional consequence of protease-substrate interactions as well as *in vivo* relevance becomes critical because not all proteolytic events may be equal in their effect. Some cleavage events may produce modest results, while others may affect critical nodes of the immune response relevant for defense against invading pathogens. Host therapies may, for example, involve reinforcing a particular immune signaling pathway for improved infection outcome (92). As a demonstration of this, a recent study investigating *Mycobacterium tuberculosis* (Mtb) and HIV coinfection highlighted potential host-directed therapies that could be developed on the basis of host protease inhibitors, identified as important players in Mtb and HIV infection (92). By resolving key host proteins integral for disease, this study demonstrates how they can be therapeutically targeted. This principle is directly translatable to the context of *S. aureus* infection, where degradomics could provide insight across the full spectrum of infection-relevant host substrates and make way for clinical intervention.

## CONCLUSION AND FINAL REMARKS

Advances in degradomic technologies have directly translated into enhanced capacity for protease substrate capture at a global level. The studies reviewed here demonstrate that global N-terminomics represents a paradigm shift in how the host-pathogen interface can be explored, enabling characterization of substrates across various biological settings and revealing a scale and complexity of host manipulation that was simply unattainable before. Furthermore, the insights gained into substrate specificity and cleavage site determination have collectively deepened our understanding of protease-substrate relationships and the molecular mechanisms that underlie their contributions to disease. Given the robust repertoire of secreted proteases *S. aureus* possesses, this bacterium serves as an ideal model for degradomic studies to analyze how these enzymes remodel the host-pathogen interface. Ultimately, degradomics reframes the challenges of therapeutic development for *S. aureus* infection, where a shift can now be afforded from ineffective antibiotic therapies toward precise interventions that target the molecular events that drive disease. To leverage the full potential of degradomic technologies, closing important gaps in the knowledge, such as the degradomic coverage of other proteases, expanding to other host niches, and functional validation, represents a critical and profound step forward in the field of staphylococcal research. Finally, the work being done on *S. aureus* degradomics may serve to inspire similar kinds of studies in other highly proteolytic bacteria, expanding the broader applicability of this approach across diverse infectious disease contexts.
